# TOO MUCH OF NOT ENOUGH: Exploring Lack of Fear and Its Consequences

**DOI:** 10.1192/j.eurpsy.2022.1900

**Published:** 2022-09-01

**Authors:** S. Jesus, A. Costa, G. Simões, G. Dias Dos Santos, M. Almeida, J. Alcafache, P. Garrido

**Affiliations:** Baixo Vouga Hospital Centre - EPE, Psychiatry And Mental Health Department, Aveiro, Portugal

**Keywords:** Physiology, Psychopathology, fear, evolution

## Abstract

**Introduction:**

Fear is an unpleasant emotional response to perceiving a threat causing physiological changes. Humans feel fear for positive motives, as it plays a crucial role in our survival. Just as the right balance in life is ideal, pathological fear is often described in one of its exaggerations, of having too much. However, lack of fear or “hypophobia” can be just as devastating and debilitating. This can be demonstrated in the analogy between those who feel no pain who also demonstrate increased risk and decreased life expectancy.

**Objectives:**

The authors aim to explore the concept of fear, discussing currently known physiological mechanisms in order to explain the effects that alterations of these mechanisms can have on fear responses, namely lack of fear, and subsequently the consequence of this on mental health.

**Methods:**

A brief non-systematized literature review was performed based on works most pertinent to the topic discussed.

**Results:**

Muted fear responses have been mentioned in the literature, principally associated with medical conditions affecting the physiological fear pathways, including Urbach-Wiethe disease. Amygdala damage provokes abnormal fear reactions and reduced fear experience. This appears to be similar to what is seen in psychopathy, where abnormalities in the limbic system produce abnormal fear responses.

**Conclusions:**

Any extreme can cause havoc on a well-balanced machine. Just as the excess of fear results in mental issues such as anxiety, a lack of fear can also be debilitating. Those demonstrating less fear could help investigators better understand mental health disorders that have been demonstrated to be mediated by similar processes.

**Disclosure:**

No significant relationships.

